# Risk Factors and Consequences of Lower Extremity Fracture Nonunions in Veterans With Spinal Cord Injury

**DOI:** 10.1002/jbm4.10595

**Published:** 2022-01-05

**Authors:** Bridget Sinnott, Cara Ray, Frances Weaver, Beverly Gonzalez, Elizabeth Chu, Sarah Premji, Mattie Raiford, Rachel Elam, Scott Miskevics, Stephen Parada, Laura Carbone

**Affiliations:** ^1^ Charlie Norwood Veterans Affairs Medical Center Augusta GA USA; ^2^ Center of Innovation for Complex Chronic Healthcare (CINCCH), Health Services Research & Development, Department of Veterans Affairs Hines VA Hospital Hines IL USA; ^3^ Parkinson School of Health Sciences and Public Health Loyola University Maywood IL USA; ^4^ Feinberg School of Medicine Northwestern University Chicago IL USA; ^5^ Department of Biostatistics University of Illinois Chicago IL USA; ^6^ Department of Mathematics Northeastern Illinois University Chicago IL USA; ^7^ Division of Rheumatology, Medical College of Georgia Augusta University Augusta GA USA; ^8^ Department of Orthopaedic Surgery, Medical College of Georgia Augusta University Augusta GA USA

**Keywords:** FEMUR FRACTURES, FRACTURE NONUNION, FRACTURE‐RELATED COMPLICATIONS, SPINAL CORD INJURY AND DISORDERS

## Abstract

We used Veterans Health Administration (VHA) national administrative data files to identify a cohort (fiscal years 2005–2014) of veterans with spinal cord injuries and disorders (SCID) to determine risk factors for and consequences of lower extremity fracture nonunions. Odds ratios (OR) for fracture nonunion were computed using multivariable‐adjusted logistic regression models. We identified three risk factors for nonunion: (i) older age (OR = 2.29; 95% confidence interval [CI] 1.21–4.33), (ii) longer duration of SCID (OR = 1.02; 95% CI 1.00–1.04), and (iii) fracture site (distal femur), with OR (comparison distal femur) including distal tibia/fibula (OR = 0.14; 95% CI 0.09–0.24), proximal tibia/fibula (OR = 0.19; 95% CI 0.09–0.38), proximal femur (OR = 0.10; 95% CI 0.04–0.21), and hip (OR = 0.13; 95% CI 0.07–0.26). Nonunions resulted in multiple complications, with upwards of 1/3 developing a pressure injury, 13% osteomyelitis, and almost 25% requiring a subsequent amputation. Our data have identified a high‐risk population for fracture nonunion of older veterans with a long duration of SCID who sustain a distal femur fracture. In view of the serious complications of these nonunions, targeted interventions in these high‐risk individuals who have any signs of delayed union should be considered. © 2021 The Authors. *JBMR Plus* published by Wiley Periodicals LLC on behalf of American Society for Bone and Mineral Research. This article has been contributed to by US Government employees and their work is in the public domain in the USA.

## 1 Introduction

Fractures are a common secondary condition for persons with a chronic spinal cord injury (hereafter referred to as spinal cord injury/disorder, or SCID), with an incident rate of 2% to 3% per year.^(^ ^1^, ^2^ ^)^ A significant subset of patients with lower extremity fractures develop post‐fracture complications, including pressure injuries, autonomic dysreflexia, pain, muscle spasms, heterotopic ossification, and nonunion.^(^ ^3^ ^)^ Fracture nonunion rates in persons with a SCID approximate 16.0%.^(^ ^4^ ^)^ Fracture nonunions are complicated pathophysiological processes that are ultimately related to problems in the biologic and mechanical environment at the fracture site.^(^ ^5^ ^)^ Prior studies of risk factors for fracture nonunion in persons with a SCID are limited by small sample sizes.^(^ ^4^ ^)^ In one report of persons with a SCID of 1 year or greater duration, fracture nonunions/delayed unions accounted for 25% of all reported post‐fracture medical complications.^(^ ^3^ ^)^ In one series of cases from Germany, fracture location (proximal femur compared with tibia), fracture management (conservative [nonoperative] compared with operative treatment), and longer duration of SCID were risk factors for fracture nonunion.^(^ ^4^ ^)^ The relationship of type of acute fracture treatment (nonoperative versus operative) to fracture nonunion is important because, in contrast with the able‐bodied population in whom management of major lower extremity fractures is almost always done operatively, treatment for lower extremity fractures in persons with a SCID remains largely nonoperative.^(^ ^6^ ^)^ To date, however, there are no published guidelines for acute fracture management in persons with a SCID.

In the able‐bodied population, long‐term consequences of fracture nonunions at the tibia include persistent pain, residual functional disability, and impaired ability to return to work.^(^ ^7^, ^8^ ^)^ However, potential consequences after fracture nonunion, including morbidities of concern to patients with a SCID, such as pressure injuries and other complications, have not been reported.^(^ ^9^ ^)^

A number of treatment modalities for fracture nonunion have been used in the able‐bodied population. These include pulsed electromagnetic fields and ultrasound,^(^ ^10^ ^)^ growth factors,^(^ ^11^ ^)^ bone stimulators,^(12^ ^)^ and medications (teriparatide).^(^ ^13^ ^)^ In some cases, however, conservative nonsurgical treatments fail, and surgical management with hardware^(^ ^14^ ^)^ and/or bone grafts^(^ ^10^ ^)^ is required. Management of fracture nonunions in persons with a SCID, however, has not previously been reported.

The primary objective of this study was to describe the predictors of fracture nonunion in persons with a SCID and to examine the relationship between initial fracture management (nonoperative versus operative) and the development of fracture nonunions. The secondary purpose was to describe the consequences of, and treatment modalities used for, fracture nonunion in persons with a SCID.

## 2 Patients and Methods

Persons with a SCID were identified from the 2016 Veterans Health Administration (VHA) Allocation Resource Center (ARC) list based on ICD‐9 codes for a SCID and treatment in either a SCID bed section and/or SCID outpatient clinic.^(^ ^15^ ^)^ ARC is a cumulative list of veterans who have ever received healthcare from a VHA facility. The ARC list was combined with healthcare utilization data from the VHA's Corporate Data Warehouse (CDW), which includes patient demographics, inpatient, outpatient, and pharmacy data. To determine SCID‐specific variables, the ARC list was linked to the VA Spinal Cord Dysfunction (SCD) Registry and/or the SCID Outcomes Database (SCIDO). The SCD Registry was an administrative database that included veterans with SCID who received care at a VHA medical facility and was used to track the population of veterans with SCID followed by each SCID Center. The SCIDO Registry continued the SCD Registry purpose; however, SCIDO also allowed clinical patient outcome data to be included. The SCD and SCIDO historical data are archived in the VHA Enterprise virtual environment. These historical data sets include information about etiology, date of onset, level of injury, completeness of injury, and the veteran's healthcare.^(^ ^15^ ^)^

Incident lower extremity fractures (International Classification of Diseases, Ninth Revision [ICD‐9] codes [820–829, 733.14, 15, 16,733.19, and 733.10]) that occurred between fiscal years 2005–2014 were identified from national VA administrative files containing utilization and diagnosis data. A fracture was considered incident if there were no encounters with the same ICD‐9 code within a 120‐day time period before the identified fracture.^(^ ^16^ ^)^ Prospective fracture nonunion cases were identified through administrative records by an ICD‐9 code of 733.82.

Nonunions coded in the 12‐month time period after the date of an incident fracture were then further examined by electronic health record (eHR) review. These records were reviewed by three physicians and two medical student extractors in a systematic fashion using a data extraction tool developed and finalized with consensus among all authors. A fracture nonunion was considered present if there was an ICD‐9 code for a fracture nonunion, a radiograph documenting lack of complete healing, and a clinical note documenting fracture nonunion. Cases without such documentation were excluded, as were those with an ICD‐9 code for nonunion and malunion on the same day. Controls were defined as those with an incident lower extremity fracture with an ICD‐9 code (820–826) with no history of a nonunion or malunion. We selected the first such incident lower extremity fracture within the data set for use in these analyses. Cases and/or controls with a date of SCID onset that was missing or after the date of incident fracture were excluded.

### 2.1 Predictors of fracture nonunion

Potential predictors of fracture nonunion considered included patient‐level characteristics (age, race, sex, body mass index [BMI]); comorbidities including diabetes mellitus, chronic kidney disease, and peripheral vascular disease;^17^, ^18^, ^19^ ^)^ and medication use (bisphosphonates, anticonvulsants, benzodiazepines, opioids, antidepressants,^(^ ^20^ ^)^ corticosteroids,^(21^ ^)^ and anticoagulants^(^ ^18^ ^)^). SCID‐related characteristics (etiology, duration, level, and extent of injury), fracture site,^(^ ^22^ ^)^ and type of fracture (open or closed^(^ ^23^ ^)^) were also considered. Patient and SCID injury level characteristics were assessed from the first time of entry into the study cohort. Comorbidities or filled medications identified during the study period at the time of or before the fracture were considered as present.

Primary treatment for the fracture was considered operative for controls if there was an ICD‐9 and a CPT code indicating an operative treatment of a fracture within 30 days of the incident fracture. Primary treatment for the fracture was considered operative for cases if eHR review identified an operative treatment within 30 days of the incident fracture resulting in nonunion. Otherwise, the treatment was considered nonoperative.

### 2.2 Complications of fracture nonunion

Complications of fracture nonunion in the first 12 months after the fracture, including pressure injuries, osteomyelitis, thrombotic events, and amputations on the same extremity as the fracture site, were identified. Only pressure injuries, osteomyelitis, and amputations that occurred at or near the fracture site were included, as determined by eHR review.

### 2.3 Treatments for fracture nonunion

Treatments for fracture nonunion, including pulsed electromagnetic fields and ultrasound,^(^ ^10^ ^)^ growth factors,^(^ ^11^ ^)^ bone stimulators,^(^ ^12^ ^)^ and medications (teriparatide, abaloperatide),^(13^ ^)^ were recorded. Whether the fracture nonunion required surgical treatment with new or revised hardware,^(^ ^14^ ^)^ amputations, and/or bone grafts^(^ ^10^ ^)^ also was identified.

### 2.4 Statistical analysis

Baseline characteristics of the study population including age (≥50 versus <50 years), race, sex, BMI (underweight, normal, overweight, and obese), comorbidities (diabetes, chronic kidney disease, peripheral vascular disease [PVD]), receipt of any prescriptions for anticonvulsants, benzodiazepines, opioids, antidepressants, anticoagulants, corticosteroids, or bisphosphonates, etiology of SCID injury (traumatic or nontraumatic), level of injury (paraplegia or tetraplegia), extent of injury (complete or incomplete), ASIA impairment scale, duration of SCID, initial treatment of fracture (operative or nonoperative), site of fracture (hip, proximal femur, distal femur, proximal tibia/fibula, or distal tibia/fibula), type of fracture (open or closed), and by whether a patient developed a fracture nonunion were examined using Pearson's chi‐square statistic or *t* test when appropriate at a level of significance set to α = 0.05. To account for multiple comparisons, a Bonferroni adjustment with adjusted α = 0.003 was considered significant.

Odds ratios (ORs) with their 95% confidence intervals (CIs) were used as the measures of association for these analyses. ORs and 95% CIs for fracture nonunion and for baseline demographics, clinical, SCID, and fracture‐related factors were estimated via a multivariable logistic regression model.

A “missing” category was created for variables in which there were missing values to retain as many observations in the analyses as possible. Because small numbers of missing fracture type (open or closed) caused problems with the logistic regression model convergence, these were imputed as closed and then open fractures.

In previous work,^(^ ^6^ ^)^ we determined that femur fractures were commonly managed with surgery as the initial fracture treatment. Thus, to mitigate confounding that might occur in the relationship between fracture treatments and nonunion if fracture site were included, in separate multivariate logistic regression models, we excluded fracture site.

Statistical analyses were conducted using SAS release 9.4 (SAS Institute, Cary, NC, USA) with two‐sided *p* values reported.

The VA Institutional Review Boards at the Charlie Norwood VA Hospital, Augusta, GA, and the Hines VA Hospital, Hines, IL, approved the study.

## 3 Results

**Fig. 1:**
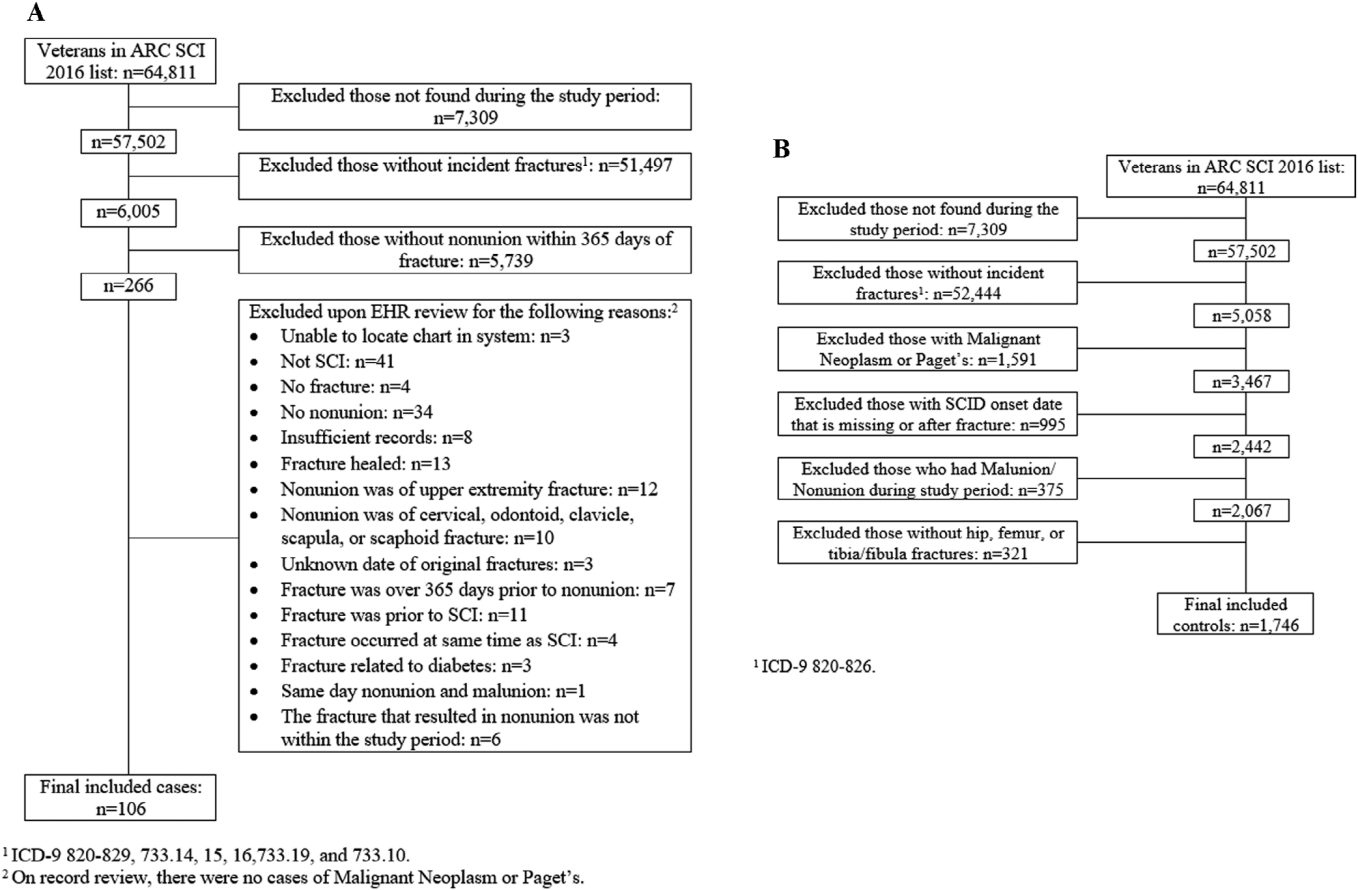
(*A*) Flow diagram of study cases. (*B*) Flow diagram of study controls.

Over the up to 10‐year time period of these analyses, there were 106 persons with at least one lower extremity fracture nonunion. All fracture nonunions occurred at the hip, femur, ankle, or tibia/fibula. Accordingly, among all eligible controls, we selected only those with an incident hip, femur, ankle, or tibia/fibula fracture. Full details regarding cohort selection and inclusion/exclusion criteria are available in Fig. 1*A* for cases and Fig. 1*B* for controls. Because the number of veterans with ankle fractures was small, ankle fractures were included with distal tibia/fibula fractures in the analyses to ensure model convergence.

Baseline characteristics of the study population with lower extremity fractures by fracture nonunion status are shown in Table 1. Persons with a fracture nonunion were more likely to be older (*p* = 0.0001), use bisphosphonates (*p* = 0.0262), have a longer duration of SCID (*p* = 0.0261), and, in sensitivity analyses in which fractures of unknown type were categorized with open fractures, to have an open fracture (*p* **<** 0.0001). Distal femur fractures were the most common fracture to sustain a nonunion (*p* < 0.0001).

**Table 1:** Baseline Characteristics of Subset of Study Population with Fracture Nonunion

Patient characteristics	Patients without fracture nonunion (*n* = 1746)	Patients with fracture nonunion (*n* = 106)	*p* Value^a^
Clinical characteristics			
Age (years), mean ± SD	57.45 ± 12.67	61.91 ± 12.01	**0.0004** ^b^
Race, *n* (%)			0.7436^c^
White	1228 (70.33)	75 (70.75)	
Black	327 (18.73)	17 (16.04)	
Other	42 (2.41)	4 (3.77)	
Missing	149 (8.53)	10 (9.43)	
Sex, *n* (%)			0.2768^c^
Male	1670 (95.65)	99 (93.4)	
Female	34 (4.35)	7 (6.6)	
Body mass index, *n* (%)			0.1738^c^
Underweight	109 (6.24)	2 (1.89)	
Normal	634 (36.31)	38 (35.85)	
Overweight	587 (33.62)	35 (33.02)	
Obese	247 (21.59)	31 (29.25)	
Missing	21 (1.2)	0 (0)	
Comorbidities, *n* (%)			0.3651^c^
Diabetes mellitus	426 (24.40)	30 (28.3)	0.9516^c^
Chronic kidney disease	129 (7.39)	8 (7.55)	0.3574^c^
Peripheral vascular disease	196 (11.23)	15 (14.15)	
Medication use, *n* (%)			
Bisphosphonates	152 (8.71)	16 (15.09)	**0.0262** ^c^
Anticonvulsants	777 (44.5)	51 (48.11)	0.4678^c^
Benzodiazepines	848 (48.57)	56 (52.83)	0.3940^c^
Opioids	1196 (68.5)	73 (68.87)	0.9368^c^
Corticosteroids	230 (13.17)	14 (13.21)	0.9918^c^
Antidepressants	940 (53.84)	58 (54.72)	0.8600^c^
Anticoagulants	273 (15.64)	24 (22.64)	0.0563^c^
SCI‐related characteristics			
Etiology of injury, *n* (%)			0.0546^c^
Traumatic	1239 (70.96)	75 (70.75)	
Non‐traumatic	470 (26.92)	25 (25.58)	
Missing	37 (2.12)	6 (5.66)	
Level of injury, *n* (%)			0.1708^c^
Paraplegia	911 (52.18)	62 (60.78)	
Tetraplegia	799 (45.76)	40 (39.22)	
Missing	36 (2.06)	4 (3.77)	
Extent of injury, *n* (%)			0.1909^c^
Complete	818 (46.85)	58 (54.72)	
Incomplete	696 (39.86)	39 (36.79)	
Missing	232 (13.29)	9 (8.49)	
ASIA impairment scale			0.0608^c^
A	509 (29.15)	41 (38.68)	
B	122 (6.99)	8 (7.55)	
C	116 (6.64)	11 (10.38)	
D	200 (11.45)	7 (6.6)	
Missing	799 (45.76)	39 (36.79)	
Duration of SCID, mean ± SD	19.71 ± 14.55	23.46 ± 15.60	**0.0103** ^b^
Fracture‐related characteristics			
Primary fracture treatment, *n* (%)			
Surgical	168 (9.62)	14 (13.21)	0.2286^c^
Non‐surgical	1578 (90.38)	92 (86.79)	
Fracture site, *n* (%)			**<0.0001** ^c^
Distal tibia/fibula	769 (44.04)	28 (26.42)	
Proximal tibia/fibula	187 (10.71)	9 (8.49)	
Distal femur	171 (9.79)	49 (46.23)	
Proximal femur	275 (15.75)	7 (6.60)	
Hip	260 (22.73)	13 (12.26)	
Fracture type; unknown type categorized as closed, *n* (%)			0.4640^c^
Closed	1714 (98.17)	103 (97.17)	
Open	32 (1.83)	3 (2.83)	
Fracture type; unknown type categorized as open, *n* (%)			**<0.0001** ^c^
Closed	1714 (98.17)	96 (90.57)	
Open	32 (1.83)	10 (9.43)	

ASIA = American Spinal Injury Association Impairment Scale; SCID = spinal cord injuries and disorders.

^a^ Bolded *p* values indicate the result was significant at the 0.05 level.

^b^ Two‐sample *t* test.

^c^ Pearson's chi‐square.

The relationship among patient demographics, clinical and SCID‐related characteristics, fracture treatment, site and type (open or closed) of fracture, and fracture treatment (operative versus nonoperative) were examined using logistic regression as risk factors for fracture nonunion. There were seven fractures of unknown type (open versus closed), all of which occurred in the nonunion cases. In multivariate analyses in which the seven fractures of unknown type were categorized as closed fractures, significant risk factors for fracture nonunion included older age (OR = 2.29; 95% CI 1.21–4.33), longer duration of SCID (OR = 1.02; 95% CI 1.00–1.04), and fracture site, such that fractures of sites other than the distal femur (reference comparator) were less likely to result in nonunion (distal tibia/fibula OR = 0.14; 95% CI 0.09–0.24, proximal tibia/fibula OR = 0.19; 95% CI 0.09–0.38, proximal femur OR = 0.10; 95% CI 0.04–0.21, hip OR = 0.13; 95% CI 0.07–0.26). In these multivariate analyses, operative initial fracture management was associated with fracture nonunion (OR = 1.91; 95% CI 1.02–3.57). In this analysis, there was a trend for open fractures to be significantly associated with fracture nonunion (OR = 2.23; 95% CI 0.67–7.43). These results from univariate and multivariate logistic regression are detailed in Table 2. Results were similar in analyses in which fractures of unknown type were categorized as open rather than closed, except that open fractures were now significantly associated with fracture nonunion (OR = 5.28; 95% CI 2.26–12.34).

**Table 2:** Predictors of Fracture Nonunion in Those With Lower Extremity Fractures

Predictors	Univariate	Multivariate
	OR (95% CI)	OR (95% CI)
Clinical characteristics		
Age (≥50 versus <50 years)	**2.23 (1.23–4.08)**	**2.29 (1.21–4.33)**
Race		
Black versus white	0.87 (0.51–1.48)	0.95 (0.54–1.67)
Other versus white	1.72 (0.63–4.73)	1.63 (0.55–4.84)
Sex (female versus male)	1.65 (0.75–3.59)	1.99 (0.82–4.88)
BMI (normal versus underweight)	2.66 (0.72–9.75)	2.51 (0.69–9.10)
BMI (overweight versus underweight)	2.65 (0.72–9.74)	2.58 (0.71–9.44)
BMI (obese versus underweight)	3.49 (0.94–12.93)	3.68 (0.99–13.74)
Diabetes mellitus (yes versus no)	1.23 (0.80–1.91)	0.90 (0.55–1.48)
Chronic kidney disease (yes versus no)	1.08 (0.52–2.23)	1.00 (0.46–2.17)
Peripheral vascular disease (yes versus no)	1.34 (0.76–2.34)	1.02 (0.56–1.86)
Anticonvulsants (yes versus no)	1.16 (0.78–1.71)	1.09 (0.69–1.73)
Benzodiazepines (yes versus no)	1.19 (0.80–1.75)	1.05 (0.68–1.62)
Opioids (yes versus no)	1.01 (0.66–1.54)	1.05 (0.64–1.71)
Antidepressants (yes versus no)	1.03 (0.70–1.53)	0.84 (0.53–1.34)
Anticoagulants (yes versus no)	**1.60 (1.00–2.56)**	1.46 (0.88–2.42)
Corticosteroids (yes versus no)	1.03 (0.58–1.83)	0.95 (0.52–1.74)
Bisphosphonates (yes versus no)	**1.91 (1.10–3.31)**	1.46 (0.80–2.66)
SCID‐related characteristics		
Etiology (nontraumatic versus traumatic)	0.89 (0.56–1.41)	1.00 (0.59–1.70)
SCI level (tetraplegia versus paraplegia)	0.74 (0.49–1.11)	0.91 (0.59–1.40)
SCI extent (incomplete versus complete)	0.79 (0.52–1.20)	1.05 (0.51–2.15)
ASIA Impairment Scale		
B versus A	0.85 (0.40–1.83)	1.23 (0.44–3.45)
C versus A	1.21 (0.61–2.41)	1.50 (0.55–4.06)
D versus A	0.46 (0.21–1.02)	0.74 (0.26–2.10)
Missing versus A	0.61 (0.39–0.95)	0.75 (0.42–1.33)
Duration of SCID	**1.02 (1.00–1.03)**	**1.02 (1.00–1.04)**
Fracture‐related characteristics		
Primary fracture treatment (operative versus nonoperative)	1.47 (0.82–2.62)	**1.91 (1.02–3.57)**
Fracture site		
Distal tibia/fibula versus distal femur	**0.13 (0.08–0.21)**	**0.14 (0.09–0.24)**
Proximal tibia/fibula versus distal femur	**0.18 (0.09–0.36)**	**0.19 (0.09–0.38)**
Proximal femur versus distal femur	**0.09 (0.04–0.21)**	**0.10 (0.04–0.21)**
Hip versus distal femur	**0.14 (0.07–0.26)**	**0.13 (0.07–0.26)**
Type of fracture (open versus closed)^a^	1.78 (0.57–5.55)	2.23 (0.67–7.43)

*Note:* Bolded odds ratio estimates and confidence intervals indicate the result was significant at the 0.05 level.

BMI = body mass index; SCID = spinal cord injuries and disorders; ASIA = American Spinal Injury Association Impairment Scale.

^a^ In the analyses detailed in this table, the 7 fractures that had unknown type (open versus closed), all of which came from those fractures resulting in nonunion, were assumed to be closed fractures.

In prespecified multivariate logistic regression models with fracture site excluded (to mitigate confounding between fracture site and initial fracture management), there was no significant relationship between type of primary fracture management and fracture nonunion (OR = 1.62; 95% CI 0.90–2.87).

Fracture nonunions resulted in a number of complications, with close to one‐third having a pressure injury, almost 25% resulting in a subsequent amputation, and approximately 13% with osteomyelitis (Table 3).

**Table 3:** Complications of Lower Extremity Fracture Nonunions

Complications (*n*, %)	Fracture nonunion
	(*n* = 106)
Pressure injury	36 (33.96%)
Osteomyelitis	14 (13.21%)
Amputation	25 (23.58%)
Thrombosis	1 (0.94%)
Other^a^	5 (4.72%)

^a^ Septic arthritis, localized osteopenia, myositis ossificans.

The most common treatment for a fracture nonunion was surgical, most frequently with new hardware. A bone stimulator was used for some nonunions (16%); pulsed electromagnetic field therapy (PEMF) was used for approximately 10% of fracture nonunions and ultrasonography was used for approximately 6%. Bone grafts and biologics were rarely used and anabolic therapies were not used at all (Table 4).

**Table 4:** Treatments for Lower Extremity Fracture Nonunions

Fracture nonunion treatment (*n*, %)	Fracture nonunion
	(*n* = 106)
Surgery	
New hardware	21 (19.81%)
Revision	8 (7.55%)
Bone graft	
Autografts	4 (3.77%)
Allografts	1 (0.94%)
Bone stimulator	
Orthofix or pulsed electromagnetic fields	11 (10.38%)
Exon ultrasonography	6 (5.66%)
Other	0
Biologics	
Bone morphogenetic protein (BMP)	1 (0.94%)
Fibroblast growth factor 2 (FGF‐2)	0
Platelet‐derived growth factor (PDGF)	0
Medications	
Teriparatide	0
Abaloparatide	0

## 4 Discussion

In veterans with a SCID, older age and longer duration of SCID were significantly associated with a fracture nonunion after a lower extremity fracture. Compared with other common lower extremity locations (ie, hip, tibia/fibula), fractures of the distal femur were most likely to incur a nonunion. Initial management of the fracture (operative versus nonoperative) was not significantly related to the development of a fracture nonunion in multivariate analyses that excluded fracture site; excluding fracture site in analyses addressing fracture management was necessary to mitigate confounding that might occur in the relationship between fracture management and nonunion by fracture site. Type of fracture (open versus closed) was not a significant risk factor for nonunion in analyses assuming those fractures with missing type were closed fractures.

Complications, most commonly pressure injuries, were frequent after fracture nonunions, with approximately one‐third of nonunions resulting in at least one complication. Surgery, including new or revised hardware, was the most common treatment for the nonunion. Bone stimulators were rarely used to treat fracture nonunions and anabolic therapies were not used at all.

There are very few published reports examining fracture nonunions in persons with a SCID and, to our knowledge, only one that has specifically examined risk factors.^(^ ^4^ ^)^ In agreement with this prior study,^(^ ^4^ ^)^ we found that longer duration of SCID was a risk factor for fracture nonunion.^(^ ^4^ ^)^ However, in contrast with this report,^(^ ^4^ ^)^ we found that older age was a significant risk factor for fracture nonunion and type of fracture treatment (operative versus nonoperative) was not.

To our knowledge, this is the first report that has examined the relationship of medication use with fracture nonunion in persons with a SCID and found no significant association of any medication examined, including anticoagulants, opioids, anticonvulsants, benzodiazepines, or antidepressants with fracture nonunion. Warfarin^(^ ^24^ ^)^ and antidepressants^(^ ^25^ ^)^ have been associated with fracture risk in elderly able‐bodied individuals, although the relationship of antidepressant use to fracture varies by antidepressant class and specific drug used.^(^ ^25^ ^)^ Opioids, benzodiazepines, and anticonvulsants are frequently used by persons with a SCID and are significantly related to fracture risk in this population.^(^ ^26^, ^27^ ^)^ That these medications were not predictive of nonunion suggests there is not a need to modify their use in post‐fracture care. This is in contrast with reports in the able‐bodied population in which opioids, benzodiazepines, and anticonvulsants have been associated with fracture nonunion.^(^ ^20^, ^28^, ^29^ ^)^ These differences may be due to differences in the pathophysiology of sublesional compared with senile osteoporosis;^30^, ^31^ ^)^ alternatively, they may reflect differences in sites of fracture.^(^ ^18^, ^28^ ^)^

In the present series, filled prescriptions for bisphosphonates also were not associated with fracture nonunion. In the able‐bodied population, the association of bisphosphonate use with fracture nonunion is controversial. Several observational studies have shown an increased risk of fracture nonunion after use of bisphosphonates.^(^ ^20^, ^29^ ^)^ However, in agreement with our findings, a randomized controlled trial of alendronate use after osteoporotic distal radius fracture with volar plate fixation in the able‐bodied population found no association between alendronate use and fracture union time.^(^ ^32^ ^)^ Other studies showed that mean union time for distal radius fractures is longer in those receiving bisphosphonates than controls but not in a clinically relevant amount,^(^ ^33^ ^)^ and no prolongation of union time at all is found at lower extremity fractures sites of femur or tibia.^(^ ^34^ ^)^ Bisphosphonates do not appear to delay fracture healing when initiated after the acute fracture, regardless of how soon after the fracture they are administered.^(^ ^35^, ^36^ ^)^ As bisphosphonates are the most frequently prescribed osteoporosis medication in persons with a SCID, prescribed to more than two‐thirds of veterans with SCID in one study,^(^ ^37^ ^)^ our study findings that pre‐fracture bisphosphonate use was not associated with lower extremity fracture nonunion in SCID is reassuring. In support of this, a forthcoming CPG (PVA Consortium for Spinal Cord Medicine Clinical Practice Guidelines: Bone Health and Osteoporosis Management in Individuals with Spinal Cord Injury) suggests that one may consider initiation of osteoporosis treatment soon after fragility fracture (data not shown).

Lower extremity fractures in the VHA system of care in veterans with a SCID are most commonly managed conservatively (ie, nonoperatively), although this is changing, and surgical management of these fractures is increasing.^(^ ^6^ ^)^ In the present study, in contrast with a prior report in which surgical management of lower extremity fractures in non‐veterans with a SCID was less likely than conservative treatment to result in a fracture nonunion,^(^ ^4^ ^)^ we found no significant association between primary treatment of the fracture and fracture nonunion. These differences may reflect the very low numbers of surgically treated fractures in our series (<10%) compared with the more than 60% managed surgically in Grassner's report.^(^ ^4^ ^)^ In agreement with our findings, Frotzler and colleagues reported that the overall rate of post‐fracture complications was similar in those treated conservatively versus surgically (15% and 13%, respectively); however, this report only included four fracture nonunions, two each in those treated surgically versus nonsurgically.^(^ ^38^ ^)^

Complications after lower extremity fractures in persons with a SCID are common, with reported rates ranging from 14% to 54%. To our knowledge, ours is the first study that has reported complications specifically related to fracture nonunion in this population. That upwards of one‐third of patients sustained a pressure injury is concerning because these can result in further functional disability, need for surgical intervention, and increased likelihood of life‐threatening infections.^(^ ^39^ ^)^ A prospective study reported that pressure injuries requiring care were associated with an almost twofold increase risk of mortality.^(^ ^40^ ^)^ In the present study, osteomyelitis developed in 13% and limb amputations in 22% of fracture nonunions. In one series, osteomyelitis was among the most common indicators for a lower extremity amputation.^(^ ^41^ ^)^ Lower extremity amputations are associated with significant functional impairment,^(^ ^42^ ^)^ major post‐surgical complications and revision amputations,^(43^ ^)^ and increased mortality.^(^ ^40^ ^)^

Fracture nonunion treatment in the able‐body population begins with examining the subtype of nonunion.^(^ ^44^, ^45^ ^)^ In support of our findings that operative treatment was the most common treatment used for fracture nonunions in persons with a SCID, operative treatment is typically recommended for fracture nonunions in the able‐bodied population. In fractures of the tibia or femur shaft, this often is addressed by placing an intramedullary nail (IMN).^(^ ^46^, ^47^ ^)^ In fractures previously treated with an IMN, it is possible to place a larger diameter IMN or to remove either the proximal or distal interlocking screws to create a dynamically locked nail instead of a statically locked nail.^(^ ^47^ ^)^ Approximately 16% of the fracture nonunions in the present study underwent treatment with a bone stimulator. Bone stimulators are used in the able‐bodied population.^(^ ^48^ ^)^ Anabolics such as teriparatide have been used off‐label to treat fracture nonunion in the able‐bodied population;^(49^ ^)^ however, there was no use of these in this SCID series. This may reflect the fact that hypercalcemia and hypercalciuria, both potential side effects of teriparatide, may be of greater concern in persons with a SCID, although this is a greater concern immediately after injury, rather than with chronic SCID.^(^ ^50^, ^51^, ^52^ ^)^

This work has a number of important strengths. To start, to our knowledge, our cohort of more than 100 fracture nonunions is larger than any prior description of fracture nonunions in persons with a SCID.^(^ ^3^, ^4^ ^)^ Furthermore, by linkage to pharmacy records, we were able to examine the relationship of medication use to fracture nonunions in persons with SCID, which has not previously been reported. This is the first report to systematically examine complications of fracture nonunions and current treatment patterns for these nonunions in persons with a SCID.

There are also several limitations to consider. In clinical practice, there is considerable variability as to what is considered a nonunion, with definitions of nonunion ranging from 2 to 12 months post fracture.^(^ ^53^ ^)^ Radiographs were not uniformly available for review; thus, OTA fracture classification was not available.^(^ ^54^ ^)^ Potentially important modifiable predictors including smoking^(^ ^23^ ^)^ and alcohol use^(^ ^20^ ^)^ were not available for the nonunion controls, as all their information was obtained solely from administrative databases. ICD‐9 codes, which were utilized in these analyses, do not include information on whether the fracture was comminuted or not. Gardner classification, which has been reported to be associated with fracture nonunion in SCID,^(4^ ^)^ was not available. Few patients underwent surgical treatment for their fracture and there may have been important differences in the association of fracture treatment (operative versus nonoperative) with the development of fracture nonunion for which we were underpowered. Additionally, while fracture nonunion in the able‐bodied population is associated with pain and delay in returns to work,^(^ ^7^ ^)^ we did not examine functional outcomes of nonunion in this study, including whether outcomes differed by ambulatory status. Future studies should examine how functional outcomes of nonunion may differ between persons with a SCI who are partially ambulatory and those who are non‐ambulatory. Regarding the relationship of medication use to nonunion, “use” of medications only represented filled prescriptions and not whether the patient actually took the medication and/or took it correctly, which is especially relevant to bisphosphonates. Because of the small number of filled prescriptions for anticoagulants other than warfarin, we were unable to examine the association of specific DOACs with the development of fracture nonunion. The association between fracture characteristics, in particular open versus closed fractures as a risk factor for fracture nonunion, deserves further study. Although it is more likely that the unknown fractures were closed rather than open fractures as lower extremity fractures in SCID are most commonly from low or no impact traumas,^(^ ^1^, ^3^ ^)^ if this was not the case, then open fractures would have been a significant risk factor for fracture nonunion in these analyses. Finally, we did not have information on stage of pressure injury.

In conclusion, older age and longer duration of SCID are key risk factors for the development of fracture nonunion in persons with a SCID. The distal femur is the most common lower extremity fracture site to develop a fracture nonunion. In view of the serious complications of these nonunions, targeted interventions in these high‐risk individuals who have any signs of delayed union should be considered.

## Disclosures

All authors state that they have no conflicts of interest.

### Peer Review

The peer review history for this article is available at https://publons.com/publon/10.1002/jbm4.10595.

## Data Availability

The data that support the findings of this study are the property of the United States Government's Department of Veterans Affairs (VA) and are only available as part of VA‐approved research activities pursuant to VHA Directives 1200.05 (2), VHA 1200.01, and/or VHA 1080.01.
